# Point‐of‐care detection of Japanese encephalitis virus biomarker in clinical samples using a portable smartphone‐enabled electrochemical “Sensit” device

**DOI:** 10.1002/btm2.10506

**Published:** 2023-04-13

**Authors:** Akanksha Roberts, Himani Dhanze, G. Taru Sharma, Sonu Gandhi

**Affiliations:** ^1^ DBT‐National Institute of Animal Biotechnology (NIAB) Hyderabad Telangana India; ^2^ DBT‐Regional Centre for Biotechnology (RCB) Faridabad Haryana India; ^3^ ICAR‐Indian Veterinary Research Institute (IVRI) Izatnagar Uttar Pradesh India

**Keywords:** diagnostics, electrochemical, JEV NS1 biomarker, point‐of‐care, screen printed carbon electrode, smartphone

## Abstract

Japanese encephalitis (JE), a neglected tropical zoonotic disease prevalent in south‐east Asian and western pacific countries, caused by the flavivirus JE virus (JEV), has a dearth of electrochemical point‐of‐care (PoC) diagnostic tools available to manage endemic breakouts. To overcome this, we have developed a screen‐printed carbon electrode (SPCE) immunosensor for rapid PoC detection of JEV nonstructural 1 (NS1) antigen (Ag), found circulating in serum of infected individuals using a smartphone based portable “Sensit” device. The modification of SPCE surface with JEV NS1 antibody (Ab) was confirmed via observation of globular protein structures via scanning electron microscopy (SEM), increase in electrode surface hydrophilicity via contact angle measurement and decrease in current via differential pulse voltammetry (DPV). The fabrication and testing parameters were optimized based on highest current output obtained using DPV. The SPCE was tested for detection limit of target JEV NS1 Ag ranging from 1 fM to 1 μM, which was determined as 0.45 fM in spiked serum. The disposable immunosensor was also found to be highly specific in detecting JEV NS1 Ag over other flaviviral NS1 Ag. Finally, the modified SPCE was clinically validated by testing 62 clinical JEV samples using both a portable miniaturized electrochemical “Sensit” device coupled with a smartphone and a laboratory‐based potentiostat. The results were corroborated with gold‐standard RT‐PCR and showed 96.77% accuracy, 96.15% sensitivity, and 97.22% specificity. Hence, this technique may further be developed into a one‐step rapid diagnostic tool for JEV, especially in rural areas.

## 1 INTRODUCTION

Japanese encephalitis virus (JEV), a flavivirus, is an endemic causing virus, which causes a neglected tropical JE disease, prevalent in south‐east Asian and western pacific countries.^1^, ^2^ The virus is transmitted to humans through the bite of *Culex* mosquitos, via a zoonotic cycle. There is currently no treatment available once infected and the available vaccination requires multiple expensive doses to be effective.^3^, ^4^, ^5^ In order to manage recurring JEV outbreaks, early‐stage sensitive detection techniques are essential to overcome conventional diagnostic limitations. These include requirement of skilled labor, expensive instrumentation, low sensitivity or specificity, high sample volume and not a rapid method. The laboratory‐based methods, which display the above listed drawbacks, include reverse transcriptase polymerase chain reaction (RT‐PCR), whole virus isolation, plaque reduction neutralization test, and hemagglutination test.^6^ Research has shown that infectious disease diagnostics^7^, ^8^, ^9^, ^10^ using electrochemical sensors is advantageous due to a lower limit of detection.^11^, ^12^, ^13^, ^14^ Furthermore, they remain unaffected by clinical sample absorbance or turbidity when compared to optical assays such as enzyme‐linked immunosorbent assays (ELISA).^15^ However, due to the required laboratory setup for signal current detection, there is an absence of on‐field electrochemical sensing devices for JEV.^16^ Currently, the only commercial point‐of‐care (PoC) diagnostic assays available for JEV are enzyme‐based immunoassays and lateral flow assay (LFA) based kits which target IgM/IgG antibodies (Ab) against JEV.^17^, ^18^ The drawback of antibody targeted detection is that Abs develop in the body after Day 4/5 of infection unlike the whole virus and/or viral antigen (Ag) present from Day 1^19^ resulting in delayed diagnosis. A recent PoC immunochromatic LFA has been developed for the detection of JEV NS1 Ag in clinically infected JEV serum samples.^20^ However, the major limitation of existing optical‐based immunoassay detection kits is the detection limit, and hence electrochemical sensors are essential for rapid detection of even trace quantities of pathogenic viral biomarkers^21^, ^22^ at early‐stage infections, for better management and therapeutic care.

Out of the three structural and seven nonstructural proteins of JEV,^23^, ^24^ we have selected nonstructural 1 (NS1) as the prospective biomarker since it is a known immunogen,^25^, ^26^, ^27^, ^28^ and found secreted in the serum of infected patients.^29^ Studies have shown that the virus takes 2–4 weeks to seroconvert in the amplifying host animal.^30^ Hence, screening for the NS1 biomarker in serum of sentinels can help in surveillance and minimize the risk of an epidemic outbreak in humans.^31^ Previously, there have been reports of ultra‐sensitive electrochemical sensors^32^, ^33^, ^34^, ^35^ developed for the detection of different JEV antigen, which include screen‐printed carbon electrode (SPCE)‐based sensors.^36^, ^37^ However, they are not portable, on‐field/bedside PoC devices, and require laboratory based detectors. The advantage of using SPCE is that precise surface area, thickness, composition (e.g., catalysts such as graphene oxide can directly be incorporated into the screen‐printing paste/ink), and accurate relative position of the three electrodes can be designed in a controlled manner.^38^, ^39^, ^40^ Moreover, in comparison to the traditional cell‐based three electrode system, the sample volume required for SPCE is minimal for electrochemical analysis.

To overcome the scarcity of bedside PoC electrochemical sensors for JEV, we have fabricated a disposable SPCE immunosensor and used a portable “Sensit” device for detection of signal currents. The “Sensit”‐based SPCE sensor has demonstrated a lower detection limit,^41^ is easier to fabricate,^33^ does not require additional signal enhancing nanomaterial,^32^, ^34^ has portable PoC field applicability,^35^ and has been validated on clinical serum samples as compared to the earlier laboratory‐based electrochemical biosensor developed for JEV diagnostics. Herein, the graphene oxide (GO) working electrode surface of the SPCE was modified with in‐house generated JEV NS1 specific polyclonal antibody as a bioreceptor using carbodiimide chemistry. The bioconjugation was confirmed via observation of globular protein structures via scanning electron microscopy (SEM), increase in electrode surface hydrophilicity via contact angle measurement and decrease in current via differential pulse voltammetry (DPV). Out of various bioreceptors applicable for protein detection^42^ such as monoclonal antibodies,^43^ aptamers^44^ and peptides,^45^, ^46^ we have selected polyclonal antibodies as they are easy to generate, less expensive, and show higher binding affinity resulting in increased sensitivity due to multiple epitope recognition for higher chances of detecting trace/mutating antigens.^47^ The fabrication and testing parameters were optimized based on highest current output obtained using DPV. The developed electrode displayed an ultra‐sensitive detection limit of 0.45 fM for JEV NS1 Ag in spiked serum ranging from 1 fM to 1 μM. Hence, the electrode can measure the minimum concentration of circulating NS1 protein required to cause infection ranging from 3.5 to 284 ng/mL as reported in similar flaviviral infections.^48^, ^49^ The SPCE sensor was designed to specifically detect JEV NS1 Ag and did not cross‐react against other closely related flaviviral NS1 Ag. It showed 3 weeks of storage stability after fabrication when stored in a dry environment at 4 °C. Finally, the modified electrode was clinically validated by testing 62 clinical JEV samples using both a portable miniaturized electrochemical “Sensit” device coupled with a smartphone and a laboratory‐based potentiostat. The results were corroborated with gold‐standard RT‐PCR and showed 96.77% accuracy, 96.15% sensitivity, and 97.22% specificity. Despite miniaturization resulting in lower range of current, the novel portable “Sensit‐”based sensor is comparable in terms of accuracy, sensitivity, and specificity to laboratory‐based electrochemical sensors and is the first electrochemical PoC sensing technique reported for JEV diagnostics with clinical sample validation. Hence, the fabricated immunosensor combined with the PoC electrochemical “Sensit” device and a smartphone may further be developed into a novel on‐site diagnostic screening assay for rapid, sensitive, and specific detection of JEV.

## 2 MATERIALS AND METHODS

### 2.1 Chemicals

SPCE with graphene oxide (GO) as the working electrode were obtained from Class One Systems (New Delhi, India). 2‐(N‐morpholino)ethanesulfonic acid (MES), Disodium hydrogen phosphate‐1‐hydrate (Na_2_HPO_4_.H_2_O), Potassium dihydrogen orthophosphate (KH_2_PO_4_), potassium chloride (KCl), and sodium chloride (NaCl) were purchased from Sisco Research Laboratories (Maharashtra, India). Sulfuric (H_2_SO_4_) and hydrochloric (HCl) acids were procured from Fisher Scientific (India). Bovine serum albumin (BSA), ethylene dichloride (EDC), and N‐hydroxysuccinimide (NHS) were purchased from Sigma‐Aldrich (India). West Nile, Dengue, and Yellow Fever viruses NS1 antigen were acquired from The Native Antigen Company (Oxfordshire, UK). JEV NS1 antigen and antibodies were generated in‐house in an earlier research experiment.^32^ Sixty‐two clinical pig serum samples validated by RT‐PCR for testing were provided by ICAR‐Indian Veterinary Research Institute (IVRI) (Bareilly, Uttar Pradesh, India). Analytical grade chemicals and solvents were used and all solutions were prepared in double distilled water.

### 2.2 Apparatus and ethics approval

Surface morphology of the working electrode of SPCE before and after fabrication was observed using a ZEISS EVO SEM (Germany) with SmartSEM software for visualization and image capture. The change in contact angle of the buffer drop on the working electrode to confirm each modification step during fabrication was recorded using a Rame‐Hart 290‐F4 goniometer (USA). DPV current changes were detected using PalmsSens4 potentiostat using PSTrace v5 desktop software and final serum samples were tested on a portable electrochemical Sensit Smart device using a smartphone PStouch v2.7 software (PalmSens, Netherlands). All experimentation was conducted at room temperature and repeated thrice unless stated otherwise. All biosafety and animal‐related protocols and procedures employed, carried out at National Institute of Animal Biotechnology (DBT‐NIAB), Hyderabad were reviewed and approved by the Institutional Biosafety Committee (IBSC) with approval number #IBSC/2019/NIAB/Sonu002 and Institutional Animal Ethics Committee (IAEC) with approval number IAEC/2019/NIAB/26/SG. The institutional and national guide for the care and use of laboratory animals was followed.

### 2.3 Fabrication and optimization of testing parameters for maximum efficiency of fabricated SPCE

In order to fabricate the SPCE, the electrode was subjected to 20 cyclic voltametric scans   with the potential range from 0.3 to 1.5 V. The SPCE surface was washed using 50 mM phosphate buffer (PB, pH 7.4), air dried and later carbodiimide activated at room temperature (RT). The activation was performed by incubating equimolar ratio of EDC:NHS (both 5 μL) on the working electrode. The electrode surface was again rinsed with PB, air dried and JEV NS1 Ab (5 μL) was added at 4°C. The unbound sites of the electrode surface were blocked using 0.1% BSA in PB (5 μL) and incubated for 1 h at RT. BSA is one of the most commonly used blocking agents as a routine practice to block excess active sites for nonspecific binding of other antigen with the electrode surface that may cause false current fluctuations.^50^ The advantages of BSA are that is a compatible protein, is inexpensive, and can be easily stored. Finally, the fabricated electrode was washed using PB, air dried and stored at 4°C in a dust‐free environment until further experimentation.

The surface morphology of the working electrode surface before and after fabrication was studied by capturing images via scanning electron microscopy at 10.00 kV and 1.6 kx magnification. Also, the increase in hydrophilicity of the electrode surface by the introduction of oxygen functionalities with each fabrication step was determined. This was done by measuring the contact angle of a water droplet on the working electrode surface at each stage using a goniometer to confirm modification. Finally, JEV NS1 Ag was added to the electrolytic buffer and allowed to bind to the immobilized Ab on the fabricated immunosensor. Electrochemical characterization of each modification and testing step was performed via DPV, and sweeping the potential from −0.5 to 0.5 V. For maximum electrode current output efficiency, fabrication and testing parameters were optimized using DPV. This included concentration of Ab coated on the working electrode surface (0.25–1.5 μg), stable response time (5–30 s), rate of each scan (0.01–0.1 V/s), buffer pH (6.0–8.0), and buffer temperature (4°C, RT, 37°C, 60°C). Following the optimized parameters, the SPCE sensors were fabricated and further tested in spiked serum.

### 2.4 Analytical performance of optimized SPCE for JEV NS1 Ag

To determine the efficiency of the fabricated SPCE, JEV NS1 Ag was spiked in serum (1 fM to 1 μM) in 10‐fold serial dilutions. The electrode was inserted into the SPCE reader of the potentiostat and 20 μL of the spiked serum samples were added. The sample was pipetted onto the SPCE ensuring that all three electrode surfaces were in contact with the liquid. The DPV was recorded in the potential range −0.5 to 0.5 V and upon plotting of the linear regression line, the limit of detection (LOD) of the modified SPCE electrochemical sensor was calculated. To validate the specificity of the SPCE sensor, 1 μM of all flaviviral JEV, West Nile (WNV), Dengue (DENV) and Yellow Fever Virus (YFV) NS1 Ag were spiked in serum and change in current was studied. The SPCE were also fabricated and stored under dry dust‐free conditions at 4°C and peak current output was measured by detecting 1 μM JEV NS1 Ag on a weekly basis for a month to confirm the storage stability of the immunosensor. Ultimately, 62 clinically validated JEV serum samples were tested on the fabricated electrode using a portable “Sensit” electrochemical device coupled with a smartphone (PStouch v2.7 software) as well as a laboratory based potentiostat (PSTrace v5 software). The results were corroborated with clinically standardized RT‐PCR to determine sensitivity of the immunosensor. JEV NS1 Ag spiked serum was used as positive control while nonspiked serum was used as negative control.

## 3 RESULTS AND DISCUSSION

### 3.1 Fabrication, working principle, and characterization of SPCE immunosensor

**FIGURE 1:**
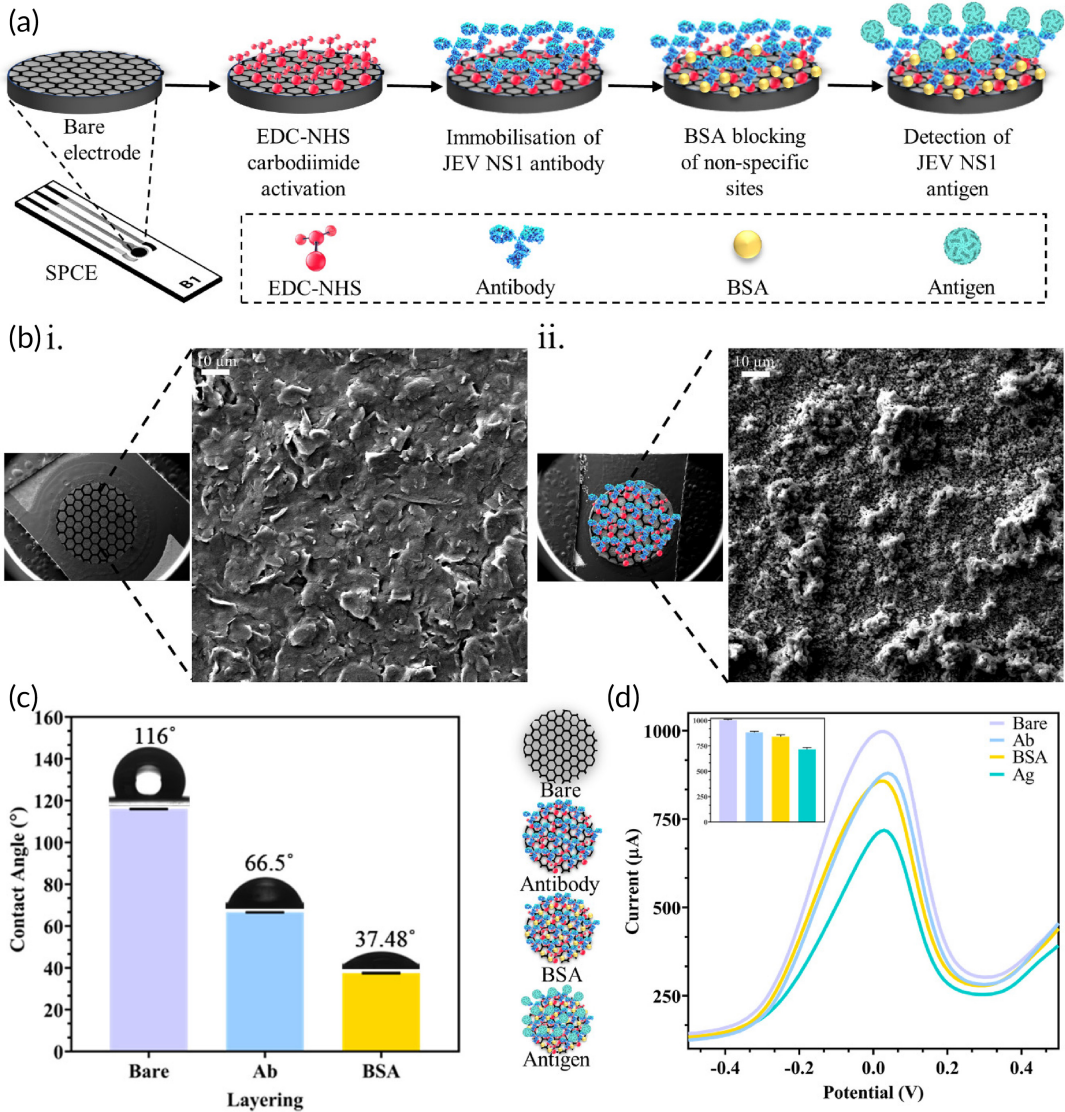
Characterization of fabricated SPCE immunosensor: (a). Sequential working electrode fabrication steps involved in the development of SPCE immunosensor; (b). SEM images depicting i. graphene layers on bare SPCE and ii. proteinaceous Ab layer deposited on fabricated SPCE; (c). Decrease in contact angle and increase in hydrophilicity of SPCE observed after each modification step; (d). DPV showing change in current output with each fabrication step with an inset bar graph displaying maximum peak current.

SPCE consists of a graphene oxide (GO) working electrode, a carbon counter electrode, and a silver reference electrode cast onto a flexible backing plastic sheet and the working electrode modification steps are demonstrated in Figure 1a. The bare working electrode surface was treated with EDC/NHS to expose the —COOH groups of graphene oxide by carbodiimide activation. EDC led to conversion of the less‐reactive carboxylic acid groups of GO into unstable O‐acyl‐urea derivatives which underwent a reaction with the NHS hydroxyl group to produce more stable intermediate esters. For immobilization of Ab bioreceptor on the activated working electrode, the Ab primary amine group formed an amide bond with the NHS ester at a physiological pH. All other activated points on the working electrode were bound by BSA protein to prevent nonspecific binding of the target Ag with SPCE. When the target Ag was allowed to interact with the fabricated sensor, the antigen–antibody binding resulted in changes in current flow, which were recorded using electrochemical detectors. SEM images depicted graphene layers before modification (Figure 1bi.) and proteinaceous antibody deposition after fabrication on the working electrode (Figure 1bii.). This confirmed change in working electrode surface morphology upon immobilization of the Ab bioreceptor onto the SPCE. Furthermore, increase in hydrophilicity of the working electrode after each modification step was observed as the wettability of the surface increased and contact angle of the water droplet decreased (Figure 1c.). The bare hydrophobic GO working electrode showed a high contact angle of 116° when a drop of water was placed on it which minimized to 66.5° upon EDC‐NHS activated Ab immobilization. In the case of Abs, the C‐terminus is a hydrophilic sequence, which enables secretion and makes them soluble in aqueous solutions.^51^ Hence, upon EDC‐NHS activation and layering of antibody on the less hydrophilic GO surface, the Abs are aligned such that the hydrophilic portion is on the upper surface, away from GO. This results in increased hydrophilicity of the working electrode. Similarly, further decrease in contact angle was observed at 37.48° between water and the BSA‐blocked electrode surface. BSA protein surface contains more hydrophilic than hydrophobic regions^52^ and upon binding to the GO surface, the exposed hydrophilic regions increased the overall hydrophilicity of the electrode surface. This increased hydrophilicity of the working electrode due to protein layering confirmed the modification steps and made the electrode ideal for target Ag detection in an aqueous‐based redox buffer environment. The change in current output which confirmed the various fabrication steps were measured via DPV after each stage as seen in Figure 1d. A decrease in current was observed upon Ab immobilization as a result of protein masking where the layer of Ab minimized the transfer of electrons from the GO surface and decreased the conducting surface. Binding of BSA during blocking to remaining unbound sites caused a slight decrease in current as the electrode conductive surface further decreased. Finally, binding of target antigen (1 μM) to the antibody bioreceptor caused a substantial minimization in peak current output due to further protein masking phenomenon.

### 3.2 Optimization of testing parameters of fabricated SPCE immunosensor

**FIGURE 2:**
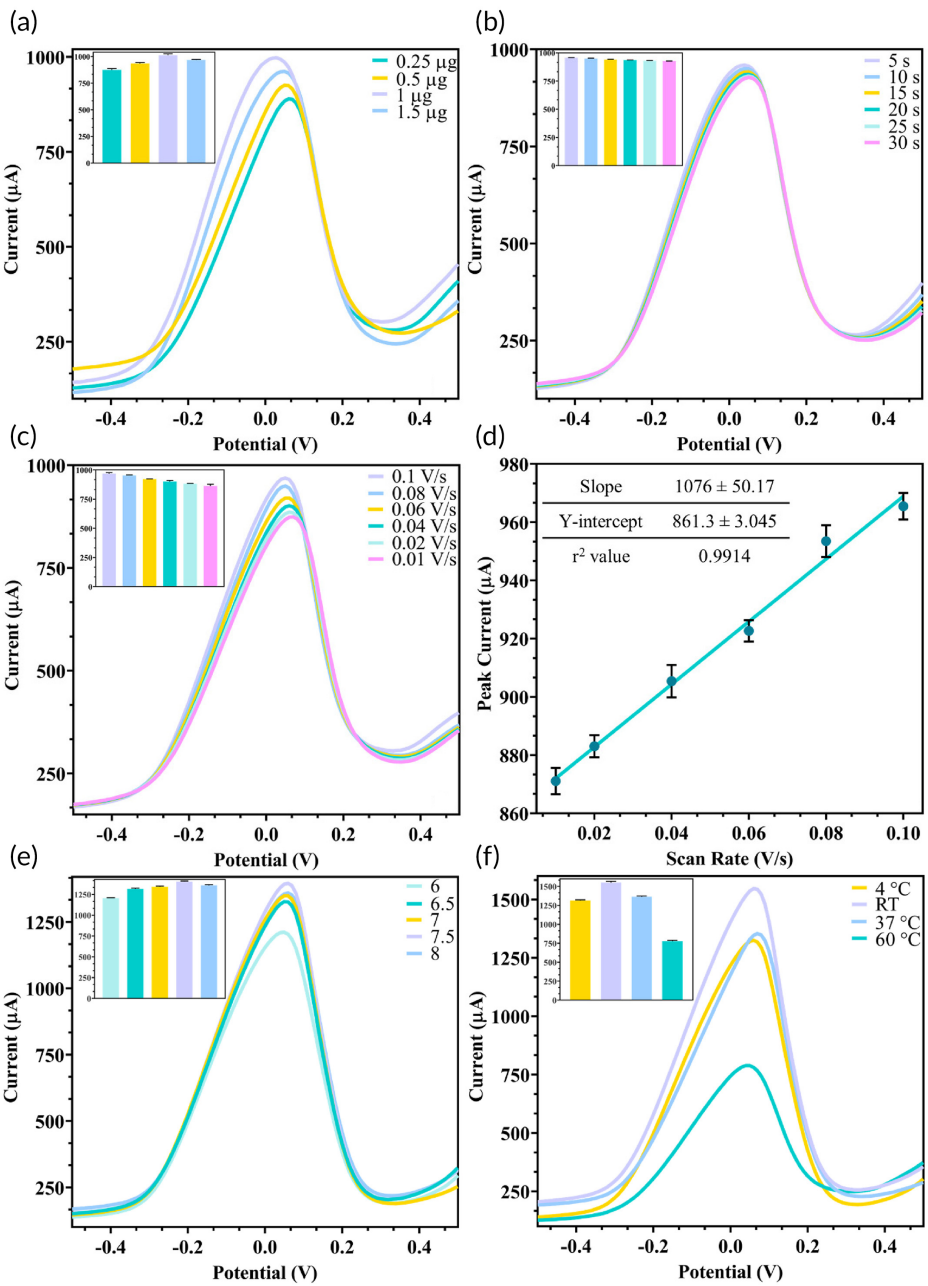
optimization of fabrication and testing parameters of developed SPCE: (a). DPV of different antibody immobilization concentrations (0.25–1.5 μg) with maximum current observed at 1 μg as displayed in the inset; (b) DPV of multiple response times and a stable reading recorded at 20 s as shown in the inset; (c) DPV of different scan rates (0.1–0.01 V/s) and highest current obtained at 0.1 V/s as depicted in the inset; (d) scan rate linear regression showed a directly proportional relation between peak current and scan rate; (e) DPV of different buffer pH (6–8) with maximum current output at physiological pH 7.5 as observed in the inset; (f) DPV of various buffer temperatures (4–60°C) with highest current output at RT as seen in the inset.

For efficient testing, various parameters were standardized for the developed SPCE by studying maximum current output via DPV. The major reason for selecting the maximum peak currents for hexacyanoferrate in DPV for all the optimization experiments is to give a maximum current range. Since the current decreases on detection of target antigen, the higher the blank current reading (i.e., more the current range), greater is the resolution to measure even a slight decrease in current upon the addition of target antigen which in turn lowers the detection limit. In case of antibody immobilization concentration, highest current was observed at 1 μg (Figure 2a) and selected as the optimum amount since beyond this concentration, the active binding sites on the electrode surface reached saturation. Upon addition of sample, the response was observed at 5 s intervals between 5 and 30 s, and a stable response was obtained at 20 s and beyond (Figure 2b), which was selected as the optimal time required for the antigen–antibody interaction to complete. The testing was also carried out at variable scan rates from 0.1–0.01 V/s and maximum current was obtained at 0.1 V/s (Figure 2c). From the linear plot of scan rate versus peak current (Figure 2d), it was found that with increasing scan rate, the current also increased. Finally, environmental testing parameters were standardized by changing the pH (6–8) and temperature (4–60°C) with most favorable results obtained at physiological pH 7.5 (Figure 2e) and room temperature (Figure 2f). The acceptable range for Ab–Ag interaction is pH 6–8^53^ as antibodies undergo fragmentation (degradation) at very low or high pH^54^ and hence the antibody used in our work showed maximum binding affinity at pH 7.5, which lies within this range. Most antibodies show maximum efficiency at either 4–8°C,^55^ room temperature,^55^ or 37°C^56^, ^57^ and tend to degrade at higher temperatures >60°C^58^ and hence the antibody used in our work showed maximum binding affinity at room temperature. The immobilized antibody bioreceptor activity showed maximum efficiency at these environmental conditions which is ideal for physiological sample testing and may be prone to degradation at other pH and temperatures resulting in loss of binding activity and hence lower current output. All further analytical experiments were carried out using the above standardized parameters.

### 3.3 Clinical validation of fabricated SPCE immunosensor

**FIGURE 3:**
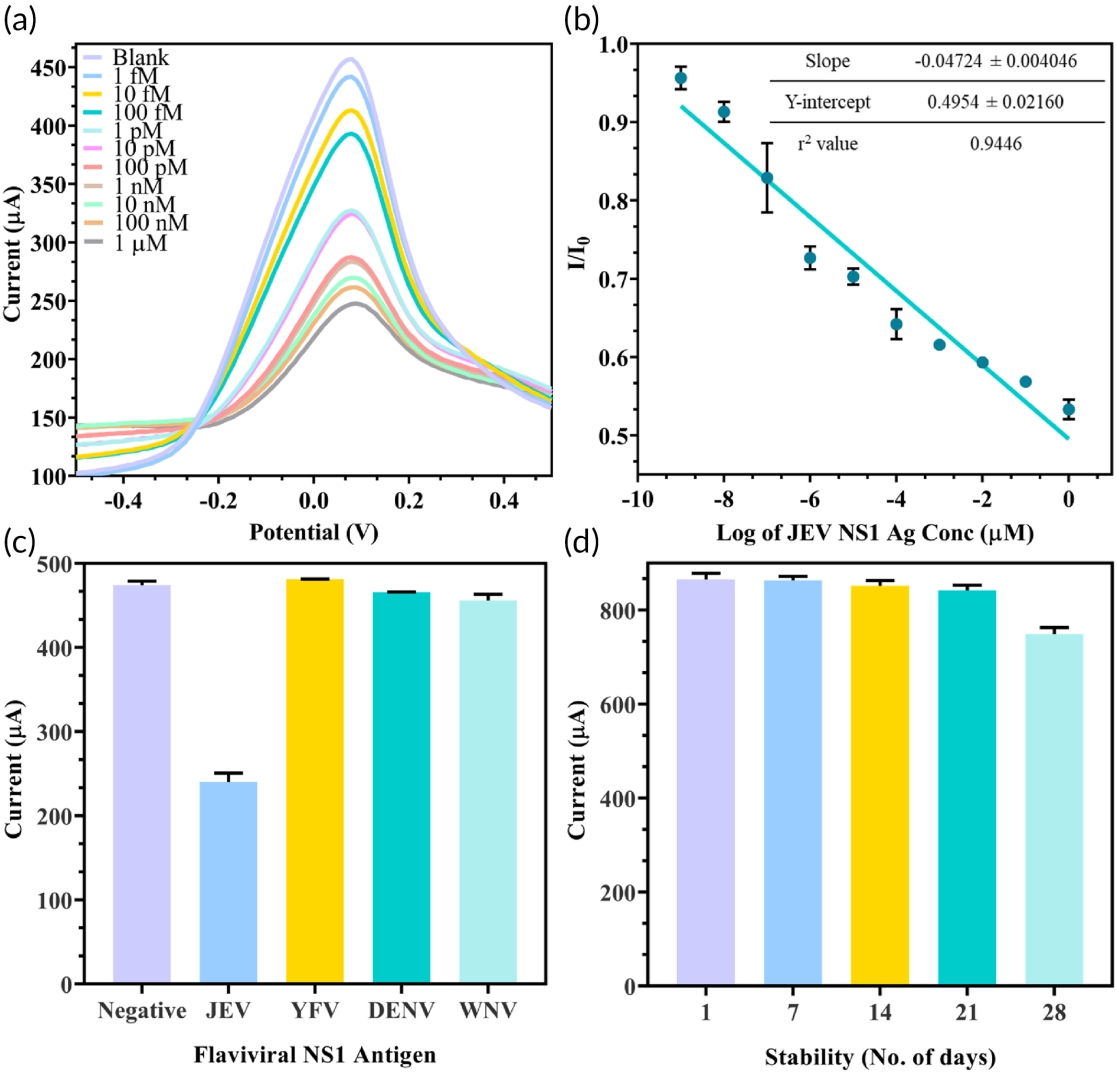
Analytical performance of the developed SPCE immunosensor: (a) DPV of increasing JEV NS1 Ag concentration in the range of 1 fM to 1 μM spiked in serum; (b) linear regression line of DPV between I/I_0_ = positive sample current/negative sample current versus log of concentration of JEV NS1 Ag in μM; (c) cross‐reactivity studies against closely related viral NS1 Ag such as WNV, DENV, and YFV against JEV; (d) storage stability analysis over a month at weekly gaps.

The analytical efficiency of the fabricated immunosensor was tested via DPV and the current was found to decrease as the amount of JEV NS1 Ag (1 fM to 1 μM) spiked in serum increased (Figure 3a). This can be explained as protein masking phenomenon as the increasing antigen layer on the electrode surface further minimized the conductive surface area, effectively minimizing surface electron transfer. To calculate the detection limit of the SPCE immunosensor, the linear regression line of Ag concentration DPV was plotted with Log of Ag concentration in μM on the x‐axis and I/I_0_ on the y‐axis where I = peak current of Ag concentration and I_0_ = peak current of negative serum (Figure 3b). The slope for the same was determined as −0.04724 ± 0.004046, y‐intercept as 0.4954 ± 0.02160, *r* ^2^ value as 0.9446 and a significant *p* value <0.0001. The *r* ^2^ value of the calibration curve of serum sample analysis in Figure 3b is 0.9446 indicating larger error and limited linearity. However, this is expected as the *r* ^2^ value is found to be lower when the Ag is spiked in biological samples such as serum in comparison to spiking the Ag in pure buffer samples^59^ due to signal interference of various other serum components. The same results upon spiking JEV NS1 Ag in pure PBS buffer would demonstrate a higher *r* ^2^ value due to no other signal interfering components.^32^ However, the *p* value of the calibration curve is significant (<0.0001), that is, the slope was significantly non‐zero and all the points on the calibration curve were statistically different from each other and hence said to be a linear regression line and the response was considered linear enough to be used for quantification. Applying the formula 3(S_y_/S), where signal/noise ratio = 3, S_y_ = standard deviation of response and S = slope of calibration curve, the limit of detection (LOD) was calculated to be 0.45 fM ranging from 1 fM to 1 μM. This LOD was highly sensitive compared to earlier JEV antigen‐based biosensors developed for JEV diagnostics as shown in Table 1.

**TABLE 1:** Comparative analysis of various JEV antigen‐based biosensors.

Type of Biosensor	Target	Limit of Detection	References
AuNPs/Co(bpy)_3_^3+^ film layer on l‐cysteine‐modified Au electrode	Whole virus	3.5 × 10^−8^ log pfu/mL	60
Bi‐layered film of AuNPs on platinum electrodes	Whole virus	6 × 10^−9^ log pfu/mL	61
Silanized interdigitated electrochemical sensor	JEV Ag	0.75 μg/mL	62
FRET based virus‐MIP fluorescent optical sensor	Whole virus	9.6 pM	63
MIP silica microspheres‐based fluorescence optical sensor	Whole virus	0.11 pM	64
Magnetic MIP‐based resonance light scattering optical sensor	Whole virus	1.3 pM	65
Electrochemical sensor using APTES‐glutaraldehyde‐serum	JEV Ag	10 ng/mL	66
PANI‐based interdigitated platinum electrochemical sensor	Whole virus	<10 ng/mL	67
PANI‐MWCNT‐modified nanocomposite microelectrode EIS sensor	JEV Ag	2 ng/mL	68
Gold coated magnetic bead‐based electrochemical sensor with MWCNT	E protein and whole virus	0.56 ng/mL 2.0 × 10 (3) pfu/mL	69
AuNP‐based SPCE electrochemical impedimetric electrochemical sensor	Whole virus	167 pfu/mL	70
AgNP‐based silanized glass slide optical sensor	JEV Ag	12.8 ng/mL	70
CNP (from starch NP)‐based SPCE electrochemical sensor	E protein	2 ng/mL	37
CNP (from chitosan NP)‐based SPCE electrochemical sensor	E protein	0.36 ng/mL	36
Graphene FET‐based electrochemical sensor	NS1 protein	1 fM	33
rGO‐based electrochemical sensor	NS1 protein	1.3 fM	32
GNR‐based electrochemical sensor	NS1 protein	0.53 fM	34
AuNPs PoC optical LFA	NS1 protein	10 pg/mL (238.1 fM)	20
SPCE PoC electrochemical sensor	NS1 protein	0.45 fM	Current work

The LOD is also lower than the minimum concentration of circulating NS1 protein reported in other flaviviral infections which ranges between 3.5 and 284 ng/mL.^48^, ^49^ Furthermore, nonspecific binding was studied by measuring the peak current of the immunosensor upon addition of 1 μM NS1 Ag of JEV, WNV, YFV, and DENV spiked in serum. It was found that while a minimization in current by 50% was observed due to binding of target JEV NS1 Ag in comparison to the negative control serum, no such decrease in current was observed for the other closely related flaviviral NS1 Ag (Figure 3c). This confirmed that the developed electrode was highly sensitive for JEV detection. Also, after fabrication, the storage stability of the SPCE was determined by read‐through the current output at 7 days intervals. It was perceived that SPCE may be stored at 4°C in a dry and dust‐free environment for up to 3 weeks without any substantial decrease in electrode performance (Figure 3d).

Finally, to validate the developed sensing system, 26 JEV positive (P1‐P26) and 36 JEV negative (N1‐N36) clinically validated serum samples (total 62) were tested on the electrode using a laboratory‐based potentiostat SPCE reader and a hand‐held smartphone based “Sensit” SPCE reader.

**FIGURE 4:**
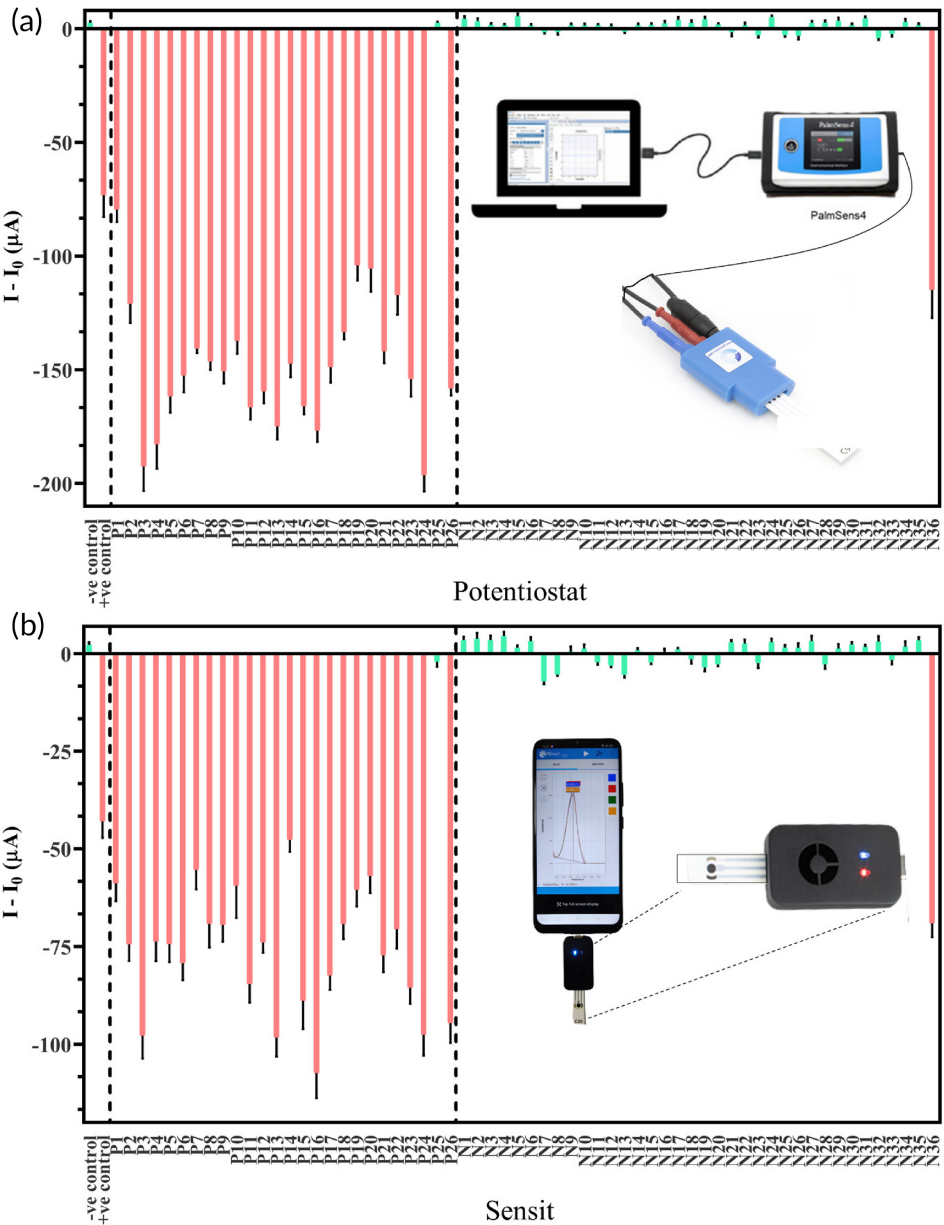
Validation of 62 JEV clinical serum samples on the fabricated SPCE: (a) laboratory‐based potentiostat and computer software; (b) miniature hand‐held “Sensit” device coupled with a smartphone and mobile application.

DPV peak current values of each SPCE before (blank) and after the addition of serum samples were recorded using PSTrace computer software for the potentiostat and PStouch mobile application for the “Sensit.” Graphs were plotted after subtracting the blank values (I_0_) from the sample values (I) and it was observed that 25 of 26 positive samples gave high negative values, which corresponded with the positive control. Similarly, 35 of 36 negative samples tended toward zero which corresponded to the negative control when tested on both the laboratory potentiostat (Figure 4a)and the portable “Sensit” device (Figure 4b). The final results were as follows: 25 true positive (TP), 35 true negative (TN), 1 false positive (FP), and 1 false negative (FN). The results have been corroborated with the laboratory standard RT‐PCR results as shown in Table 2, and accuracy (TP + TN/TP + TN + FP + FN), sensitivity (TP/TP + FN) and specificity (TN/TN + FP) of the fabricated immunosensor were determined as 96.77%, 96.15%, and 97.22%, respectively.^71^, ^72^ While the current range of the potentiostat is greater by 50–100 μA as compared to the “Sensit” due to miniaturization of the device, it however, does not interfere with the final results. Hence, the hand‐held portable smartphone‐based “Sensit” using fabricated SPCE may be developed into a novel electrochemical PoC diagnostic tool for JEV.

**TABLE 2:** Comparative analysis of clinical samples using the portable “Sensit,” laboratory‐based potentiostat and gold standard RT‐PCR.

S. No.	Sample label	Sample code	RT‐PCR	Potentiostat	Sensit
1	P1	1	Positive	Positive	Positive
2	P2	7538G	Positive	Positive	Positive
3	P3	7543M	Positive	Positive	Positive
4	P4	7545G	Positive	Positive	Positive
5	P5	7557M	Positive	Positive	Positive
6	P6	7557G	Positive	Positive	Positive
7	P7	9279	Positive	Positive	Positive
8	P8	9292	Positive	Positive	Positive
9	P9	9258	Positive	Positive	Positive
10	P10	9776	Positive	Positive	Positive
11	P11	9777	Positive	Positive	Positive
12	P12	9173	Positive	Positive	Positive
13	P13	9287	Positive	Positive	Positive
14	P14	9296	Positive	Positive	Positive
15	P15	9805	Positive	Positive	Positive
16	P16	9286	Positive	Positive	Positive
17	P17	10664	Positive	Positive	Positive
18	P18	10676	Positive	Positive	Positive
19	P19	8666	Positive	Positive	Positive
20	P20	8652	Positive	Positive	Positive
21	P21	8663	Positive	Positive	Positive
22	P22	8658	Positive	Positive	Positive
23	P23	8660	Positive	Positive	Positive
24	P24	8972	Positive	Positive	Positive
25	P25	9032	Positive	Negative	Negative
26	P26	9038	Positive	Positive	Positive
27	N1	1	Negative	Negative	Negative
28	N2	2	Negative	Negative	Negative
29	N3	3	Negative	Negative	Negative
30	N4	4	Negative	Negative	Negative
31	N5	5	Negative	Negative	Negative
32	N6	6	Negative	Negative	Negative
33	N7	9298	Negative	Negative	Negative
34	N8	9210	Negative	Negative	Negative
35	N9	9204	Negative	Negative	Negative
36	N10	9188	Negative	Negative	Negative
37	N11	9282	Negative	Negative	Negative
38	N12	9302	Negative	Negative	Negative
39	N13	9303	Negative	Negative	Negative
40	N14	9297	Negative	Negative	Negative
41	N15	9166	Negative	Negative	Negative
42	N16	9305	Negative	Negative	Negative
43	N17	7912	Negative	Negative	Negative
44	N18	8656	Negative	Negative	Negative
45	N19	8659	Negative	Negative	Negative
46	N20	8651	Negative	Negative	Negative
47	N21	8662	Negative	Negative	Negative
48	N22	10505	Negative	Negative	Negative
49	N23	10518	Negative	Negative	Negative
50	N24	9020	Negative	Negative	Negative
51	N25	9023	Negative	Negative	Negative
52	N26	9029	Negative	Negative	Negative
53	N27	9018	Negative	Negative	Negative
54	N28	9010	Negative	Negative	Negative
55	N29	9039	Negative	Negative	Negative
56	N30	9005	Negative	Negative	Negative
57	N31	9001	Negative	Negative	Negative
58	N32	9035	Negative	Negative	Negative
59	N33	9040	Negative	Negative	Negative
60	N34	8996	Negative	Negative	Negative
61	N35	8941	Negative	Negative	Negative
62	N36	8991	Negative	Positive	Positive

The highlighted the row 25 and 62 as the outcome of the results were different than the RT‐PCR, so it will be easy to differentiate.

## 4 CONCLUSION

To overcome the dearth of PoC electrochemical diagnostic techniques for JEV, we have fabricated and optimized a SPCE biosensor for rapid, sensitive, and on‐site mass screening of early‐stage JEV. The electrode was able to detect JEV NS1 Ag in serum with an LOD of 0.45 fM. The SPCE immunosensor showed minimal nonspecific binding against other closely related cross‐reactive viral NS1 and storage stability after fabrication up to 3 weeks at 4°C. With a response time of 30 s and minimal sample preparation, this electrochemical sensor can be used for rapid mass screening. Lastly, the SPCE biosensor was able to successfully detect the NS1 Ag using a hand‐held portable smartphone‐based “Sensit” electrochemical device with 96.77% accuracy, 96.15% sensitivity, and 97.22% specificity. Despite miniaturization resulting in lower range of current, the novel portable “Sensit”‐based sensor is comparable in terms of accuracy, sensitivity, and specificity to laboratory‐based electrochemical sensors and is the first electrochemical PoC sensing technique reported for JEV diagnostics with clinical sample validation. The main limitation/drawback of the proposed method is repeatability due to current variations observed from one blank SPCE to another, which may further be standardized by optimizing the electrode printing, fabrication, and modification steps. This may be carried out by using different types of conductive material ink and painting/printing techniques^40^ and selecting the one with consistent current output. Clinical serum samples can directly be added to the electrode and readings taken immediately on‐site using a hand‐held portable smartphone‐based “Sensit” electrochemical device without the need to elaborate laboratory instruments. Hence, this detection technique shows future potential to be developed into a one‐step PoC diagnostic tool for JEV, especially for application in rural endemic prone areas, which lack extensive laboratory setups for early‐stage mass screening and better management of JEV.

## AUTHOR CONTRIBUTIONS

**Akanksha Roberts:** Data curation (lead); methodology (lead); validation (equal); writing – original draft (lead); writing – review and editing (equal). **Himani Dhanze:** Validation (supporting). **Sonu Gandhi:** Conceptualization (lead); funding acquisition (lead); project administration (lead); supervision (equal); writing – review and editing (lead). **G. Taru Sharma:** Funding acquisition (supporting).

## CONFLICT OF INTEREST STATEMENT

The authors have no conflicts of interest to declare.

## Data Availability

The data that support the findings of this study are available from the corresponding author upon reasonable request due to privacy/ethical restrictions.
